# Purification and Structure Characterization of the Crude Polysaccharide from the Fruiting Bodies of *Butyriboletus pseudospeciosus* and Its Modulation Effects on Gut Microbiota

**DOI:** 10.3390/molecules28062679

**Published:** 2023-03-16

**Authors:** Run Tian, Lu-Ling Wu, Hong-Fu Li, Zhi-Qun Liang, Pei-Hu Li, Yong Wang, Nian-Kai Zeng

**Affiliations:** 1 Key Laboratory of Tropical Translational Medicine of Ministry of Education, School of Pharmacy, School of Tropical Medicine, College of Biomedical Information and Engineering, Hainan Medical University, Haikou 571199, China; 2 School of Pharmacy, North China University of Science and Technology, Tangshan 063210, China; 3 College of Science, Hainan University, Haikou 570228, China

**Keywords:** crude polysaccharide from *Butyriboletus pseudospeciosus* (BPP), physicochemical properties, functions, gut microbiota, Tax4Fun

## Abstract

Polysaccharides from the species of Boletaceae (Boletales, Agaricomycetes, Basidiomycota) are economically significant to both functional foods and medicinal industries. The crude polysaccharide from *Butyriboletus pseudospeciosus* (BPP) was prepared, and its physicochemical properties were characterized through the use of consecutive experimental apparatus, and its impact on the gut microbiota of Kunming mice was evaluated. Analyses of the structure characteristics revealed that BPP was mainly composed of Man, Glc, and Gal, possessing the pyranose ring and β/α-glycosidic linkages. TG analysis exhibited that BPP had great heat stability. The SEM observation performed demonstrated that BPP appeared with a rough, dense, and porous shape. Through the BPP intervention, the serum and fecal biochemical index in mice can be improved obviously (*p* < 0.05). The abundance of beneficial microbiota in the BPP-treated group was significantly increased, while the abundance of harmful microbiota was significantly decreased (*p* < 0.05). Based on the Tax4Fun, we also revealed the relationship between the species of gut microbiota and showed that the high dose of BPP has significantly changed the functional diversities compared with those in other groups (*p* < 0.05). The results suggest that *B. pseudospeciosus* could serve as potential functional food or medicine.

## 1. Introduction

Boletaceae (Boletales, Agaricomycetes, Basidiomycota) is a large family characterized by an umbrella-shaped fruiting body with dense tubes, occasionally with lamellae. It is distributed widely in the temperate, subtropical to tropical regions of the world [1,2,3,4]. In China, some species of Boletaceae were treated as medicinal fungi in “South Yunnan Medica”, and their efficacies included expelling pathogenic heat, enriching weakness, refreshing the brain, and improving blood circulation. Pharmacological studies have revealed that Boletaceae has antidiabetic, anticancer, immunoregulatory, and antioxidative activities [5,6,7]. Currently, the species of Boletaceae are regarded as promising functional food, and they have underwent an increasing number of investigations due to their delicious taste and versatile nutrients [8]. A variety of bioactive components of Boletaceae have been documented, such as polysaccharides, phenols, terpenoids, alkaloids, and sterols.

Polysaccharide, as an aplenty substance in fungi of Boletaceae, has been recognized to generate pharmacological effects in several experimental studies. For example, *Boletus edulis* was proved to have antitumor effects since the polysaccharide restrains the proliferation of breast cancer cells (MDA-MB-231 and Ca761) and promotes apoptosis induced by mitochondrial pathways [9]. The polysaccharide of *Leccinum duriusculum*, in turn, exerts the immunoregulation ability by reducing the secretion of NO and immune factors of RAW 264.7 (IL-6 and TNF-α) [10]. Meanwhile, the polysaccharide from *B. aereus* also exhibits great antitumor activity and protects the immune organs [11]. Although other polysaccharides from fungi such as ganoderan [12], lentinan [13], and grifolan [14] have beneficial effects on gut microbiota, the effect of polysaccharides from boletes on regulating gut microbiota remains to be elucidated.

*Butyriboletus pseudospeciosus* is a species of Boletaceae originally described from Yunnan Province, China [15], which has drawn increasing attention due to its edibility. Herein, the crude polysaccharide from the fruiting bodies of *B. pseudospeciosus* (BPP) is isolated, and its physicochemical properties are characterized. Moreover, the effects of BPP on the gut microbiota were studied.

## 2. Results and Discussion

### 2.1. Species Identification

The voucher specimen (FHMU1391) was discovered to be clustered with the holotype of *B. pseudospeciosus*, with strong support (BS = 97%, PP = 1.00) (Figure S1). Moreover, our collections also matched well with the protologue of *B. pseudospeciosus* [3]. The morphological and phylogenetic evidence confirmed that our sample is *B. pseudospeciosus.*

### 2.2. Chemical Composition of BPP

**Figure 1:**
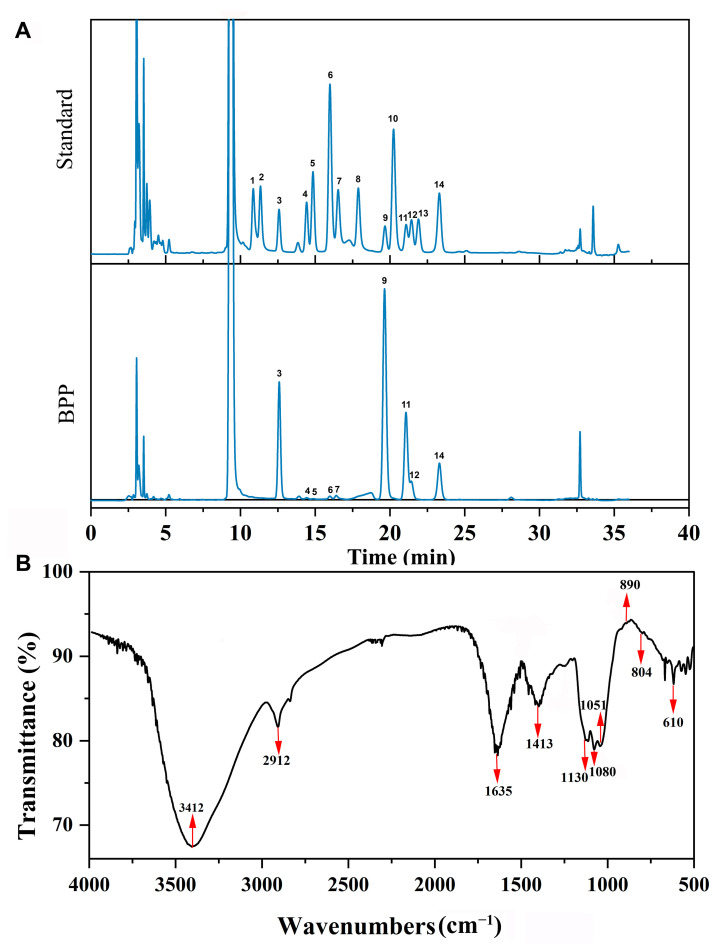
Structural characterization of BPP. (**A**) Fingerprint chromatograms of the monosaccharide compositions of BPP and fourteen kinds of reference monosaccharides. 1. GulA. 2. ManA. 3. Man. 4. Rib. 5. Rham. 6. GlcN. 7. GlcA. 8. GalA. 9. Glc. 10. GalN. 11. Gal. 12. Xyl. 13. Ara. 14. Fuc. (**B**) FTIR spectrum of BPP.

The crude polysaccharide from *B. pseudospeciosus* was prepared, and it was 4.20% relative to the dried BPP samples (100 g). The contents of carbohydrates, protein, and uronic acid in BPP were 64.00%, 1.95%, and 1.19%, respectively (Table 1). The HPLC detection suggested that glucose (Glc), mannose (Man), and galactose (Gal) were the most abundant components in the compositions of BPP (Figure 1A), which is in line with the previous studies [7]. The results manifest that the polysaccharides from the fungi of the family Boletaceae could be composed of Glc, Man, and Gal mostly.

### 2.3. FTIR Analysis of BPP

Fourier-transform infrared spectroscopy (FTIR) was used to analyze the glycosidic bond and functional groups of BPP [16]. BPP shared a strong absorption peak at 3412 cm^−1^ and a weak absorption peak at 2912 cm^−1^, which were assigned to the stretching peaks of O-H and C-H, respectively (Figure 1B). The intense absorption peak approximately at 1635 cm^−1^ could arise from the carbonyl group of the polysaccharide [7]. The absorption peak detected at 1413 cm^−1^ was due to the C-H variable angle vibration. Additionally, the absorptions at 1130 cm^−1^, 1080 cm^−1^, and 1051 cm^−1^ may be correlated to C-O-C and C-O-H stretching peaks, which are consistent with the pyranose ring structure [17]. The absorption peaks at 890 cm^−1^ and 804 cm^−1^ suggested the presence of *β*- and *α*-glycosidic bonds in BPP, respectively [18], whereas the peaks observed at 610 cm^−1^ could relate to the skeletal modes of pyranose [19].

### 2.4. NMR Spectroscopy Analysis

The ^1^H-NMR spectrum of BPP is shown in Figure S2A. Previous reports have reported that the crowded and narrow regions in the range of 3.0–5.5 were typical signals of polysaccharides [7,20], which are in line with our spectrum. As shown in Figure S2A, the anomeric hydrogen signals at 5.11 and 4.93 can be observed, respectively. Then, the anomeric hydrogen signals lower than 5 ppm corresponded to the *β*-pyranose unit mostly, while greater than 5 ppm corresponded to the *α*-pyranose unit [17,21], which suggests that BPP contains both *α*-pyranose and *β*-pyranose sugar units. The resulting spectrum is consistent with previous studies relating to the polysaccharides from the species of Boletaceae [7].

As shown in Figure S2B, we can find three carbon signals in the anomeric region (δ 103.51, 103.02, and 97.77) in the ^13^C NMR spectrum. In the previous documents [22,23], the chemical shifts in the pyranose residues in the ^13^C NMR spectrum were in the range of δ 100–106 for the *β*-configuration and δ 93–100 for the *α*-configuration. Thus, the results revealed that BPP contains both *β*-pyranose and *α*- pyranose. In addition, given that the uronic acid content is low, the absorption peak at δ 179 could relate to the presence of acetyl and ester group within polysaccharide by combining with Figure S3. The HSQC spectrum demonstrated the correlations between anomeric ^13^C signals and ^1^H signals. As shown in Figure S4, three cross peaks of δ_H/C_ 4.93/98.1, 4.84/103.3, and 4.61/104.1 were observed in the HSQC spectrum, which confirmed the presence of *β*-configuration and *α*-configuration in the BPP [24].

### 2.5. Thermal Stability of BPP

The thermal stability of the crude polysaccharide was detected using thermogravimetric (TG) analysis. BPP underwent a slight weight loss (14.62%) around 131.5 °C (Figure S5), probably owing to the evaporation of free water and bound water. The initial thermal degradation temperature of BPP was up to around 200 °C, suggesting that BPP maintains good thermal stability. When the temperature increased from 200 °C to 562 °C, the weight loss reached a maximum of 83.39%.

### 2.6. Scanning Electron Microscope (SEM) Analysis

**Figure 2:**
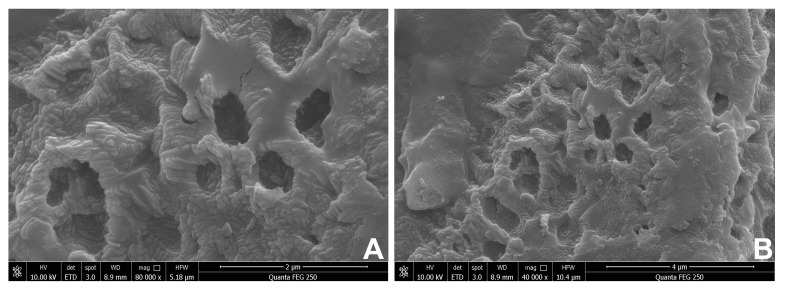
SEM images of BPP. (**A**) Magnification at 80,000×. (**B**) Magnification at 40,000×.

SEM was extensively used to detect the surface morphological characters of BPP [25]. The surface of BPP was rough, and it exhibited a dense and porous shape, as observed by SEM (Figure 2), indicating it may benefit the application of BPP as drug carriers [26,27]. The microstructure of the BPP was different from those of other mushrooms, such as *Ganoderma lucidum*, *Hericium erinaceus*, and *Calocybe indica.* The polysaccharide of *G. lucidum* has entirely intact and compact structures in a spongy form, *H. erinaceus* polysaccharide exhibited flakiness with smooth surfaces, and the *C. indica* polysaccharide has a smooth surface and no discernible network [28,29,30].

### 2.7. BPP Influences Viscera Weight

Compared with the K group (spleen index: 0.19 ± 0.01; thymus index: 0.86 ± 0.10), the spleen index and thymus index of the other groups increased, but these indexes in the PH (0.26 ± 0.03%, 1.11 ± 0.08), G (0.26 ± 0.03, 1.11± 0.22), and PHG (0.27 ± 0.04, 1.12 ± 0.19) were significantly higher than those in the K group (*p* < 0.05). There was no significance in the liver index among the groups except for the PHG group, and the liver index of all mice increased across all times (Table 2).

### 2.8. Biochemical Analyses of Feces and Serum

The pH value of the treatment groups decreased gradually from 0 to 2 weeks. Meanwhile, a significant pH decrease was observed in PL, PH, G, PLG, and PHG (*p* < 0.01) (Table 3). Therefore, BPP exhibited a great power to decrease the pH value in both low and high doses groups, which is in accordance with the previous literature that plantain polysaccharide could reduce the pH value when in vitro fermented [31]. A significant decrease in the ammonia content in the PL and PH groups was found compared with the K group (Table 3), which indicates that BPP can reduce the ammonia content in the gut. The biochemical analyses are presented in Table 4. In the treatment groups, the total content of diamine oxidase (DAO) decreased significantly (*p* < 0.01) compared with the K group. Thus, we can speculate that BPP improves gut health by reducing the gut pH value. BPP can significantly decrease the DAO and ET content in the serum. Traditionally, it has been argued that ammonia, as a microbial metabolite in the gut, had potential poisonous effects on host health [32], and the amount of the DAO and ET in the serum inversely correlates with the permeability and integrity of the intestine [33,34].

### 2.9. BPP Influences Gut Microbiota

#### 2.9.1. BPP Influences Gut Microbiota Diversity

In the present study, the influence of BPP on the gut microbiota was examined using the bacterial 16S rRNA sequence. A total of 4,695,001 pairs of reads were obtained from the sequences of the fecal samples, with an average of 104,333 reads per sample. The average length of all sample sequences was mainly between 410 and 430 reads, which indicates that our results meet the requirements. The sample dilution curve (Figure S6A) and rank abundance curve (Figure S6B) tended to be gentle, indicating that the sampling is sufficient and our data volume maintains large enough for subsequent analysis. The flower diagram of OTUs analysis indicated that a total of 318 OTUs were contained in all experimental groups. Twenty-four unique OTUs were in the K group, 11 in the G group, 28 in the PL group, 11 in the PH group, 23 in the PLG group, and 3 in the PHG group, respectively (Figure S7). Therefore, the OTUs value of PL, PLG, and K groups are similar, indicating the microbiota compositions of these three groups are similar. The OTUs values of the G, PH, and PHG groups are similar and lower than that of the K group, which suggests that high-dose administration of BPP could reduce the number of species of gut microbiota.

**Figure 3:**
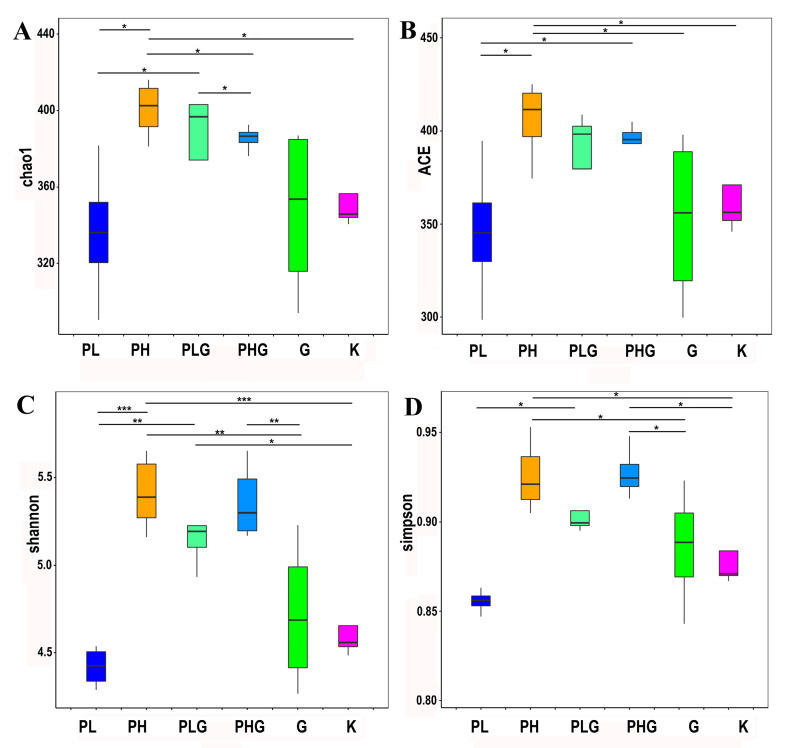
Comparison of microbial diversity between the different groups in serum using different measures of *α*-diversity. (**A**) Chao one (**B**) ACE. (**C**) Shannon. (**D**) Simpson. * *p* < 0.05, ** *p* < 0.01, and *** *p* < 0.001.

The alpha diversity was presented in our studies (Figure 3). The gut microbiota diversity could be assessed by alpha diversity analysis, including the Chao one, ACE, Shannon, and Simpson index [35]. The results demonstrated that the Chao one and ACE indexes of PH, PLG, PHG, and G are higher than those of the K group, but only PH showed a significant difference from the K group (*p* < 0.05). Therefore, it can be inferred that the diversity of the gut microbiota was significantly changed under the high dose of BPP treatment.

**Figure 4:**
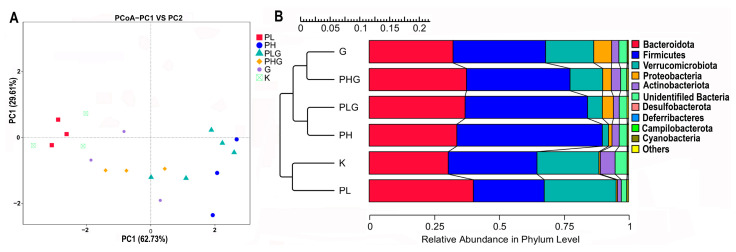
(**A**) Comparison of bacterial composition among all groups by PcoA at OTU level. (**B**) Clustering tree of UPGMA.

Principal coordinated analysis (PCoA) was used to study the distinctions of microbiota for each group. The two principal axes demonstrated a 92.34% variation (PC1: 62.73% and PC2: 29.61%) (Figure 4A). The microbiota of the PH and PLG groups exhibited a longer distance from the PL, PHG, and G groups, suggesting that the PH and PLG groups have a greater effect on the gut microbiota diversity. The gut microbiota of the PL and K groups are similar, suggesting that the low dose of BPP does not markedly change the diversity of the gut microbiota. In addition, the Unweighted Pair–group Method with Arithmetic Mean (UPGMA) analysis demonstrated that the PH and PLG groups clustered together, farther away from the K group (Figure 4B). The UPGMA analysis is in accordance with the result of the PCoA analysis, indicating that the PH and PLG groups could change gut microbiota diversity.

#### 2.9.2. BPP Influences Gut Microbiota Composition

**Figure 5:**
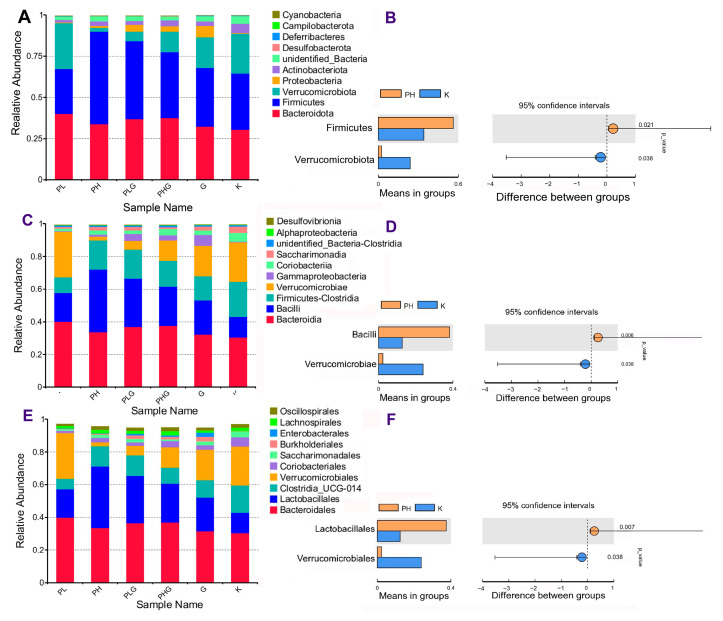
Taxonomy analysis of microbiota at a different level about BPP on gut microbiota. (**A**) Clustering of top 10 species with different treatments at the phylum level. (**B**) Differences between PH and K at the phylum level. (**C**) Clustering of top 10 species with different treatments at the class level. (**D**) Differences between PH and K at the class levels (**E**) Clustering of top 10 species with different treatments at the order level. (**F**) Differences between PH and K at the order level.

The influences of BPP on the gut microbiota composition at the phylum, class, order, family, genus, and species levels were studied. The gut microbiota composition of the PH group was obviously changed compared with the K group. At the phylum level (Figure 5A), *Firmicutes*, *Bacteroidota*, *Proteobacteria*, and *Verrucomicrobiota* were the most abundant types of gut microbiota in the K group, which are consistent with the previous study [36]. After 2 weeks of high dose of BPP treatment, the relative abundance of *Firmicutes* increased significantly from 33.75% to 56.20% (*p* < 0.01), while the relative abundance of *Verrucomicrobiota* decreased significantly from 23.93% to 2.35% (*p* < 0.01) (Figure 5B). The ratio of *Bacteroidota*/*Firmicutes* decreased obviously compared with the K group. Previous reports have shown that the polysaccharides could ameliorate chronic pancreatitis by reducing the ratio of *Bacteroidota*/*Firmicutes* and improve DSS-induced ulcerative colitis by enhancing the abundance of *Firmicutes* [37,38].

At the class level (Figure 5C), the abundance of *Bacilli* in the PH group (38.19%) was significantly higher than that of the K group (12.69%) (*p* < 0.01), while the abundance of *Verrucomicroiae* in the PH group (2.35%) was significantly lower than that of the K group (23.93%) (*p* < 0.05) (Figure 5D). Additionally, at the order level (Figure 5E), the gut microbiota of the PH group consisted of *Bacteroidales*, *Lactobacillales*, *Clostridia UCG-014*, and *Verrucomicrobiales*. *Lactobacillales*, which accounted for 12.41% of the K group, drastically increased to 37.48% in the PH group (*p* < 0.01) (Figure 5F). *Verrucomicrobiales* dramatically decreased from 23.93% (in the K group) to 2.35% (in the PH group) (*p* < 0.05). Additionally, Li et al. [39] also found that the TC (total cholesterol), TG (triglyceride), and LDL-C (low-density lipoprotein cholesterol) in HFD-induced SD rats could be reduced by inhibiting some microbiota, such as *Verrucomicrobiales*.

**Figure 6:**
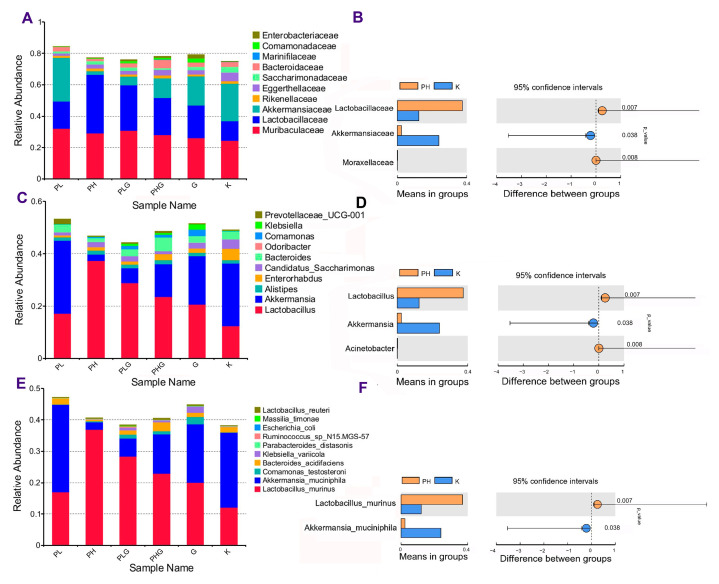
Taxonomy analysis of microbiota at a different level about BPP on gut microbiota. (**A**) Clustering of top 10 species with different treatments at the family level. (**B**) Differences between PH and K at the family level. (**C**) Clustering of top 10 species with different treatments at the genus level. (**D**) Differences between PH and K at the genus levels. (**E**) Clustering of top 10 species with different treatments at the species level. (**F**) Differences between PH and K at the species level.

At the family level (Figure 6A), the gut microbiota of the PH group mainly consisted of *Lactobacillaceae*, *Muribaculanceae*, and *Akkermansiaceae*. As can be seen in Figure 6B, the abundance of *Lactobacillaceae*in in the PH group (37.42%) was significantly higher than that of the K group (12.40%) (*p* < 0.01), while the abundance of *Akkermansiaceae* in the PH group (2.35%) was significantly lower than that of the K group (23.93%) (*p* < 0.05). It was noted that the proliferation of *Lactobacillaceae* in the gut could be associated with the treatment of obese diabetics, DSS-induced colitis, and colorectal cancer in mice [40,41,42].

At the genus level (Figure 6C), the abundance of *Lactobacillus* and *Acinetobacter* in the PH group increased significantly (*p* < 0.01), while the abundance of *Akkermansia* decreased significantly (*p* < 0.05) (Figure 6D) compared with the K group. Increasing evidence has revealed that *Lactobacillus,* a kind of key probiotic, exerts benefits to ulcerative colitis and protects the cardiovascular system by resisting pathogens or modulating cytokine secretion [43,44].

At the species level (Figure 6E), the gut microbiota of the treatment groups is mainly composed of *Akkermansia muciniphila* and *Lactobacillus murinus.* It can be seen from Figure 6F that the abundance of *L. murinus* increased significantly from 12.10% (in the K group) to 36.94% (in the PH group) (*p* < 0.01), while the abundance of *A. muciniphila* decreased significantly from 23.93% (in the K group) to 2.35% (in PH group) (*p* < 0.05). The previous report demonstrated that *Lactobacillus murinus* could be used as a useful probiotic to improve animal health and reduce the risk of gastrointestinal disorders [45], indicating that BPP could maintain intestinal health by improving the proliferation of *L. murinus*.

In conclusion, we have analyzed the influences of all treatments on the gut microbiota at different levels, and the high dose of BPP has significantly changed the microbial communities in the gut compared with other treatment groups (*p* < 0.05). Therefore, BPP could have a potential prebiotic potency by modulating the composition and abundance of the gut microbiota.

#### 2.9.3. BPP Influences Gut Microbiota Functions

**Figure 7:**
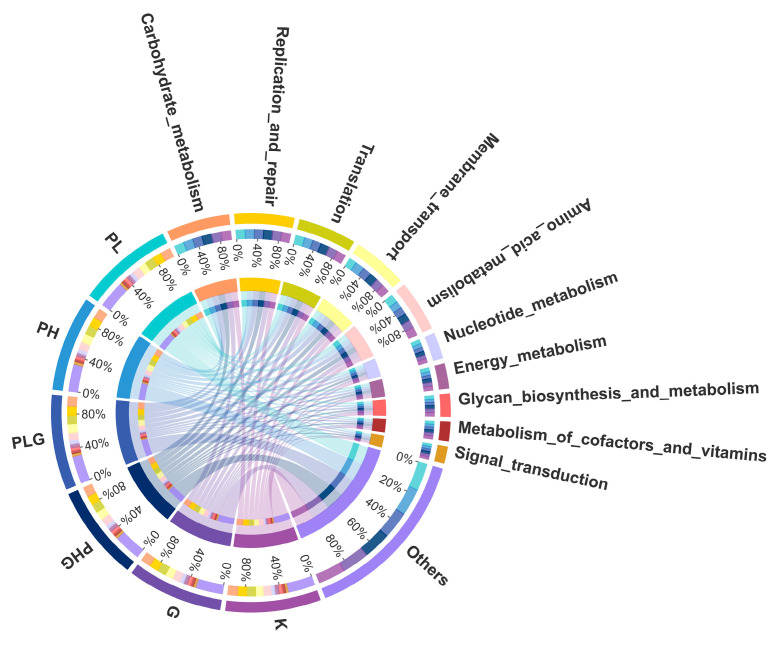
KEGG metabolic pathways at the second level.

**Figure 8:**
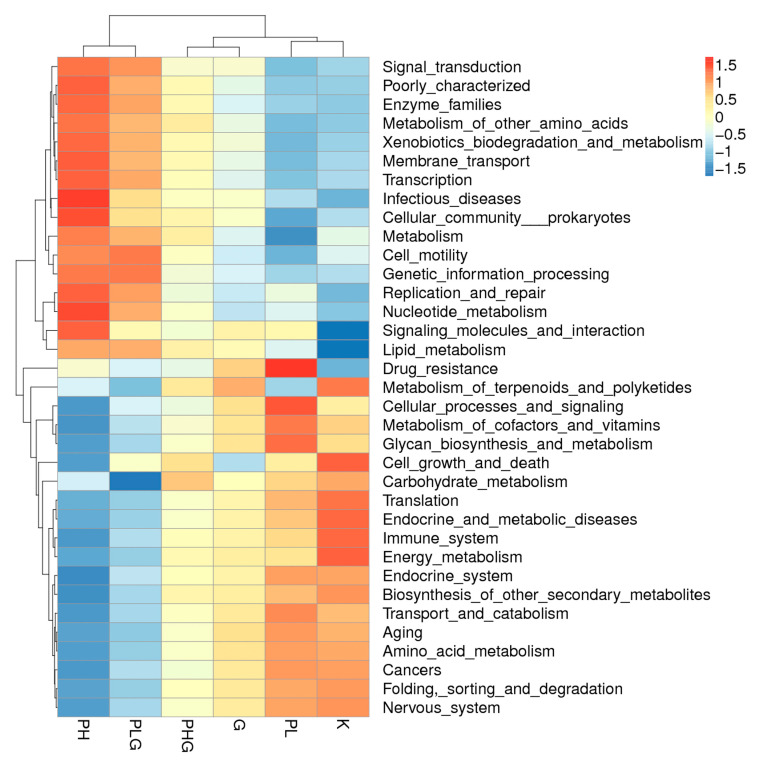
Functional prediction picture of Tax4Fun between different groups in mice.

Tax4Fun was implemented to predict the gene content of the gut microbiota to demonstrate the varied functional diversities [46]. A total of 35 metabolic pathways were annotated, of which signal transduction, metabolism of co-factors and vitamins, glycan biosynthesis and metabolism, energy metabolism, nucleotide metabolism, and amino acid metabolism were the six most abundant pathways (Figure 7). Moreover, in the functional analyses (Figure 8), the high dose of BPP significantly changed the functional diversities and metabolism pathways more than that of the other groups (*p* < 0.05). The BPP treatment regulated gut microbiota functions, indicating that a high dose of BPP may impose potential and beneficial effects on human diseases by regulating the gut microbiota. In addition, the high dose of BPP obviously upregulated the genes that were responsible for lipid metabolism, nucleotide metabolism, membrane transport, translation, and xenobiotics biodegradation and metabolism (*p* < 0.05), which is similar to previous reports [47,48], suggesting that BPP could regulate the metabolism of the gut microbiota. Collectively, functional predictions via Tax4Fun demonstrate that BPP may modulate the gut micrology by regulating those bacteria to change their metabolites and cellular processes.

## 3. Materials and Methods

### 3.1. Materials and Chemicals

The fruiting bodies of the mushroom used in this study were purchased from the market of Kunming, Yunnan Province of China. The voucher specimen (FHMU1391) was preserved in the Fungal Herbarium of Hainan Medical University (FHMU), Haikou City, Hainan Province, China. Techniques of specie identification, including morphological and molecular phylogenetic analyses, followed the methods of Zeng et al. [2], Chai et al. [3], and Chai et al. [49]. Three DNA sequences [nuc 28S rDNA D1-D2 domains (28S) (MH879687), nuc translation elongation factor 1-*α* gene (*TEF1*) (MH879716), and internal transcribed spacer 1 and 5.8S ribosomal RNA gene (*ITS*) (MH885349)] from the voucher specimen were deposited in GenBank.

Diamine oxidase (DAO) and endotoxin (ET) assay kits were purchased from Mlbio Technology Co., Ltd. (Shanghai, China). Monosaccharide standard samples were all bought from Sigma-Aldrich (Steinheim, Germany); other chemicals were of analytical grade and mostly manufactured by Xilong Scientific Co., Ltd. (Guangdong, China).

### 3.2. Preparation of the Crude Polysaccharide

The dried fruiting bodies of *B. pseudospeciosus* were ground into powder (40 meshes). Then, 100 g of powder was extracted with boiling water at a ratio of 1:25, followed by an ultrasonic-assisted extraction at 50 °C for 15 min and extracted with hot water at 85 °C for another 3 h. The above procedure was repeated twice. After extraction, the sample solution was centrifuged for 15 min at 4000 r/min; the combined filtrate was concentrated to one-fourth of its initial volume with a rotary evaporator under decompressed pressure at 50 °C and then deproteinized with Sevag reagent. Finally, the crude polysaccharide of *B. pseudospeciosus* was obtained by a four-fold volume of 95% ethanol precipitation, dialysis (molecular weight cutoff at 3.5 kDa) in distilled water, and vacuum lyophilization. The total carbohydrate, protein, and uronic acid content of the polysaccharide were determined by Tian et al. [7] and Yang et al. [50].

### 3.3. Monosaccharide Composition Analysis

The monosaccharide composition was measured using high-performance liquid chromatography (HPLC) equipped with a DAD detector. The column was C_18_ column (250 mm × 4.6 mm, 5 µm, Agilent 1100, Santa Clara, CA, USA), the injection volume was 5 µL, and the flow rate was 1 mL/min. Mobile phase A was 100 Mm PBS buffer (pH = 6.7), and mobile phase B was acetonitrile. The detection wavelength was set at 250 nm, and the column temperature was maintained at 30 °C.

### 3.4. Infrared Spectrum (IR) Analysis

Dried BPP (1 mg) and KBr powder (100 mg) were mixed and ground to make the pellet. FT-IR was used to assess the absorption spectrum of the sample with the wavenumber range of 4000–400 cm^−1^.

### 3.5. Thermal Stability of BPP

The thermal gravimetric (TG) analyses of BPP were conducted using a TGA/DSC3 + instrument (Mettler Toledo, Zurich, Switzerland). The BPP samples were placed on the Al_2_O_3_ crucible under an air atmosphere. Additionally, the temperature was increased from 25 °C to 800 °C at 10 K/min.

### 3.6. NMR Spectroscopy Analysis

BPP (60 mg) was dissolved in deuterium oxide (D_2_O) and frozen at −20 °C for 24 h, followed by thawing at room temperature, and the above procedure was performed three times. Then, the samples were re-dissolved with D_2_O and placed in an ECZ400S 400 MHz NMR instrument (JEOL, Tokyo, Japan) to measure the ^1^H NMR,^13^C NMR, DEPT-135, and HSQC spectra at room temperature.

### 3.7. Scanning Electron Microscope (SEM) Analysis

The dried BPP was coated with gold, and its morphological features were recorded by a Quanta FEG 250 SEM system (FEI, Columbia, SC, USA) at the accelerating voltage of 10.0 kV.

### 3.8. Animal Study

**Figure 9:**
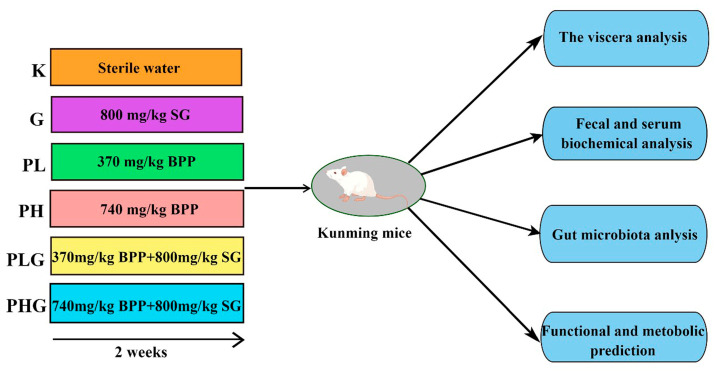
Schematic diagram of experimental design. The figure was drawn by Figdraw [https://www.figdraw.com (accessed on 2 June 2022)].

All of the experimental procedures were followed in accordance with the guidelines for the Care and Use of Laboratory Animals of the National Institutes of Health. Kunming mice were purchased from Skbex Biotechnology Co., Ltd. (Anyang, China), license number SCXK (Yu) 2020-0005, all of which were male, 6–8 weeks old, and weighing 20–30 g. The mice were housed in a temperature-controlled environment (20–25 °C) with a 12 h daylight circle. The mice were adapted to the environment for one week and could freely take in standard chow and sterilized water. After acclimation for one week, the mice were randomly divided into six groups (*n* = 7 per group): the normal group (K), the low dose (370 mg/kg) of the BPP group (PL), the high dose (740 mg/kg) of BPP group (PH), the positive (800 mg/kg spore powder of *G. lingzhi*) group (G), the (370 mg/kg of BPP + 800 mg/kg spore powder of *G. lingzhi*) group (PLG), and the (740 mg/kg of BPP + 800 mg/kg spore powder of *G. lingzhi*) group (PHG). BPP and the spore powder of *G. lingzhi* were dissolved in distilled water, and each group was administered via intragastric administration (0.3 mL). The mice in the normal group were supplied with the same volume of distilled water. After two weeks of feeding, all of the animals fasted for 8–12 h. On the 15th day, the serum samples were prepared from mice blood collected from the eye sockets, followed by centrifuge at 4000 r/min for 8 min. The fresh feces in the colon were collected and snap-frozen with liquid nitrogen until use. The liver, spleen, and thymus were excised and weighed, and the organ index was measured by the following formula: organ index (%) = organ weight/body weight × 100%. The schematic graph of the experimental procedure is shown in Figure 9.

### 3.9. Serum and Feces Biochemical Analysis

ELISA kits (Mlbio Technology Co., Ltd., Shanghai, China) were used to quantitate the diamine oxidase (DAO) and endotoxin (ET) levels in the serum. Additionally, the ammonia concentration of feces was measured according to the description given by Joseph et al. [51]. Additionally, the pH values were determined by Jin et al. [52].

### 3.10. Fecal Flora Genomic DNA Extraction and Amplicon Sequencing

The genomic DNA from the fecal flora of each group was extracted following the instructions of the IMBS DNA Extraction Kit (TIANGEN, Beijing, China), and the purity and concentration of the extracted DNA were detected by 2% (*w*/*v*) agarose gel electrophoresis. Total genomic DNA was processed by the Servicebio Technology Co., Ltd. (Wuhan, China), and it was followed as described by Sang et al. [12]. In brief, V3–V4 variable regions of the bacterial 16S rRNA gene were amplified by PCR with specific primers (515F and 806R). Subsequently, the PCR amplification products were homogenized and quantified, and they were purified by Qiagen Gel Extraction Kit (Germantown, MD, USA). Sequencing libraries were constructed and loaded using TruSeq DNA PCR-Free Sample Preparation Kit from Illumina (San Diego, CA, USA) according to the manufacturer’s instructions and sequenced with Illumina NovaSeq 6000 platforms.

### 3.11. Data Analysis and Bioinformatics

The reads of each sample were merged and assembled by Fast Length Adjustment of SHort reads (FLASH) software [V1.2.7, http://ccb.jhu.edu/software/FLASH (accessed on 18 November 2020)], and the given sequence was called Raw Tags. Then, the high-quality tag data (Clean Tags) was obtained by using the Quantitative Insights Into Microbial Ecology (QIIME) software (V 1.9.1) to filter the spliced Raw Tags. These clean tags were compared with the reference database to detect the chimera sequences that were removed, and then effective tags were obtained for the subsequent bioinformatics analysis.

Sequence analyses were displayed by Uparse software (V7.01001), and sequences with ≥ 97% similarity were assigned to the same operational taxonomic units (OTUs), in which representative sequence was screened to obtain homologous species information and abundance distribution. The abundant group of each sample at all levels, including phylum, class, order, and family, were analyzed. Both QIIME software (1.9.1) and R software (2.15.3) were used to analyze the sample diversity (*α* and *β*-diversity). Tax4fun was used to predict the functions of the microbiota, and the predicted genes and their functions were aligned to the Kyoto Encyclopedia of Genes and Genomes (KEGG) database.

### 3.12. Statistical Analysis

All of the statistical analyses were conducted using SPSS 16.0 software. The data were obtained as mean ± SEM (standard error of the mean), and the one-way ANOVA was used to compare the groups to each other. Significance was recorded at *p* < 0.05 and *p* < 0.01 levels. Additionally, other diagrams were drawn by Origin 2021 V9.8.0.

## 4. Conclusions

The crude polysaccharide of *B. pseudospeciosus* (BPP) was prepared and characterized in this study. BPP is a dense, porous polysaccharide with a pyranose ring that exhibits good heat stability. BPP could increase the thymus and spleen index, indicating that BPP could be used as a potential immunoregulation food. BPP can reduce the intestinal pH value, which may be due to the production of short-chain fatty acids (SCFAs). The lower pH value in the gut could facilitate the proliferation of beneficial bacteria. Thus, BPP has an effective modulation effect on the gut environment by inhibiting ammonia release and alleviating the production of DAO and ET. A high dose of BPP (PH) and a low dose of BPP combined with the G (PLG) treatments could both influence gut microbiota diversity. Furthermore, the high dose of BPP (PH) can increase the beneficial bacteria, such as *Lactobacillus.* The high dose of BPP can obviously upregulate the genes that are responsible for lipid metabolism, nucleotide metabolism, membrane transport, translation, and xenobiotics biodegradation and metabolism. Therefore, BPP could have a beneficial effect by modulating the diversity, composition, and function of gut microbiota.

## Figures and Tables

**Table 1:** Chemical compositions of BPP.

Chemical Composition (*w*/*w*, %)	
Carbohydrate	64.00
Protein	1.95
Uronic acid	1.19
**Monosaccharide Component (%)**	
Mannose (Man)	17.12
Ribose (Rib)	0.16
Rhamnose (Rham)	0.018
Glucosamine(GlcN)	0.11
Glucuronic acid(GlcUA)	0.38
Glucose (Glc)	54.87
Galactose (Gal)	20.58
Xylose (Xyl)	3.02
Fucose (Fuc)	3.82

**Table 2:** Viscera index of mice with different treatments.

Groups	Liver (%)	Spleen (%)	Thymus (%)
K	4.42 ± 0.33	0.19 ± 0.01	0.86 ± 0.10
G	4.85 ± 0.62	0.26 ± 0.03 *	1.11 ± 0.22 *
PL	4.47 ± 0.62	0.24 ± 0.05	1.11 ± 0.08 *
PH	4.50 ± 0.46	0.26 ± 0.03 *	1.12 ± 0.17 *
PLG	4.45 ± 0.49	0.25 ± 0.04	1.12 ± 0.24 *
PHG	5.16 ± 0.44 *	0.27 ± 0.04 *	1.12 ± 0.19 *

* *p* < 0.05.

**Table 3:** pH values and ammonia content of mice feces with different treatments.

Groups	pH Value	Ammonia Content (μg/g)
K	8.32 ± 0.07	44.72 ± 4.13
G	8.22 ± 0.09 *	40.38 ± 0.66 **
PL	8.23 ± 0.05 *	41.75 ± 0.66 *
PH	8.18 ± 0.01 **	41.43 ± 0.27 *
PLG	8.04 ± 0.05 **	42.89 ± 0.47
PHG	8.03 ± 0.09 **	40.89 ± 0.11 *

* *p* < 0.05, ** *p* < 0.01.

**Table 4:** Content of D-LA and ET in the serum of mice with different treatments.

Groups	DAO (pg/mL)	ET (EU/mL)
K	430.56 ± 34.69	29.43 ± 1.34
G	234.41 ± 32.52 **	30.70 ± 1.40
PL	322.75 ± 24.97 **	26.69 ± 3.75 *
PH	339.30 ± 26.34 **	23.13 ± 1.74 **
PLG	333.08 ± 29.43 **	37.70 ± 2.04 **
PHG	319.35 ± 37.83 **	37.11 ± 1.06 **

* *p* < 0.05, ** *p* < 0.01.

## Data Availability

The data presented in the study are deposited in the https://www.ncbi.nlm.nih.gov/GenBank (accessed on 1 September 2022), accession number of GenBank (28S: MH879687; TEF1: MH879716; and ITS: MH885349).

## Supplementary Materials

The following supporting information can be downloaded at: https://www.mdpi.com/article/10.3390/molecules28062679/s1, Figure S1: Phylogram of *Butyriboletus* inferred from a mulTILOcus (rDNA 28S, ITS, *tef1*, and *RPB2*) dataset using RAxML. [BS (≥50 %) and PP (≥0.95) are indicated above the branches]; Figure S2: NMR spectra of BPP. (**A**) ^1^H NMR spectrum of BPP. (**B**) ^13^C NMR spectrum of BPP; Figure S3: DEPT-135 spectra of BPP; Figure S4: HSQC spectra of BPP; Figure S5: TG analysis of BPP under atmosphere at a heating rate of 10 K/min; Figure S6: KEGG metabolic pathways at the second level; Figure S7: The flower figure OTUs distribution of flora with different treatments.

## Sample Availability

Samples of the compounds are not available from authors.
