# Supplementary material for: Purification and Structure Characterization of the Crude Polysaccharide from the Fruiting Bodies of Butyriboletus pseudospeciosus and Its Modulation Effects on Gut Microbiota
Source: Molecules. 2023 Mar 16;28(6):2679. doi: 10.3390/molecules28062679 (PMC10057200; doi:10.3390/molecules28062679)
Supplement: Supplementary file 1 [file molecules-28-02679-s001.zip › molecules-2256754-supplementary.pdf]

Supplementary Materials

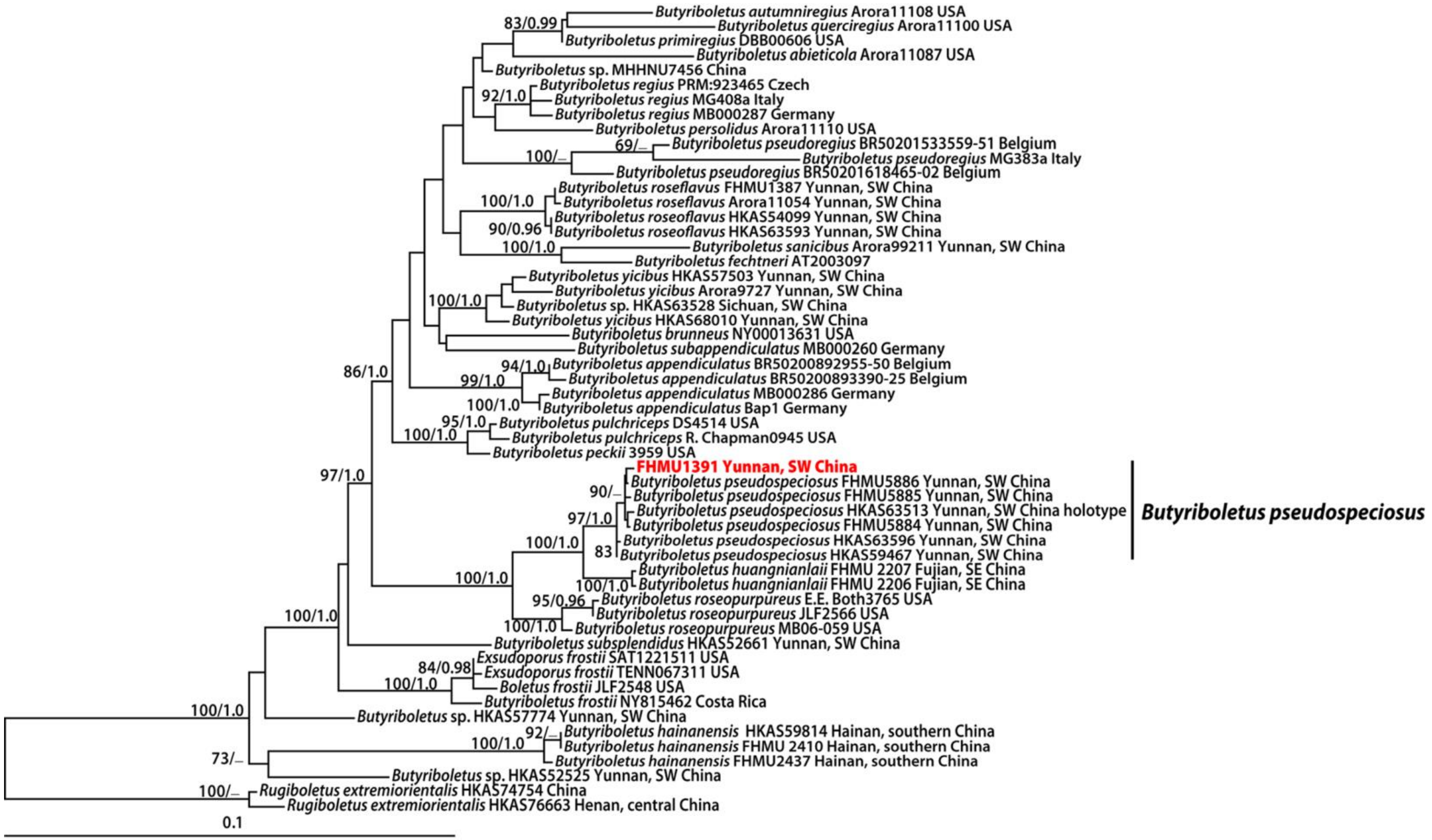

Figure S1: Phylogram of Butyriboletus inferred from a mulTILOcus (rDNA 28S, ITS, tef1, and RPB2) dataset using RAxML. [BS (≥50 %) and PP (≥0.95) are indicated above the branches]

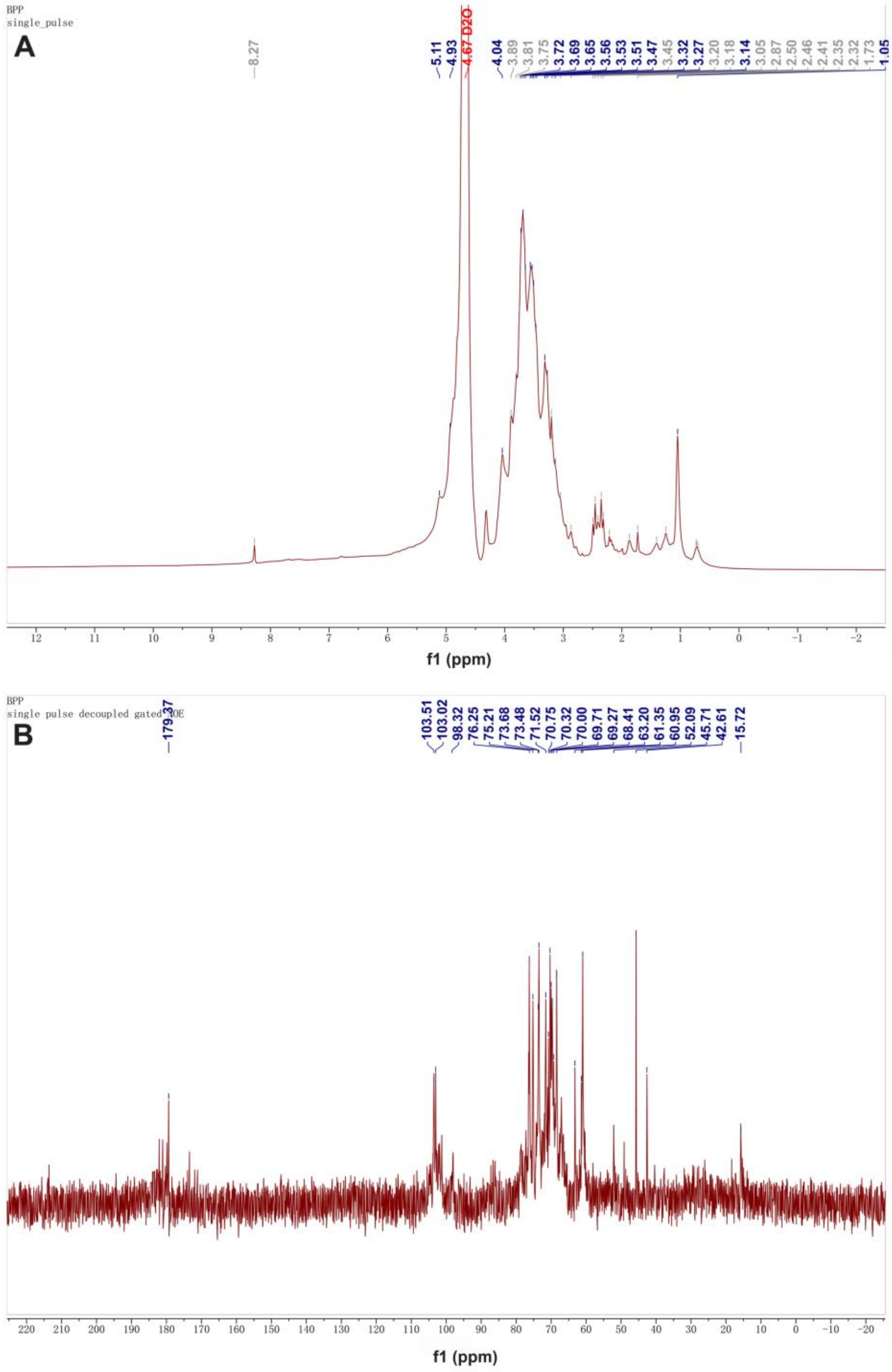

Figure S2: NMR spectra of BPP. (A) 1H NMR spectrum of BPP. (B) 13C NMR spectrum of BPP

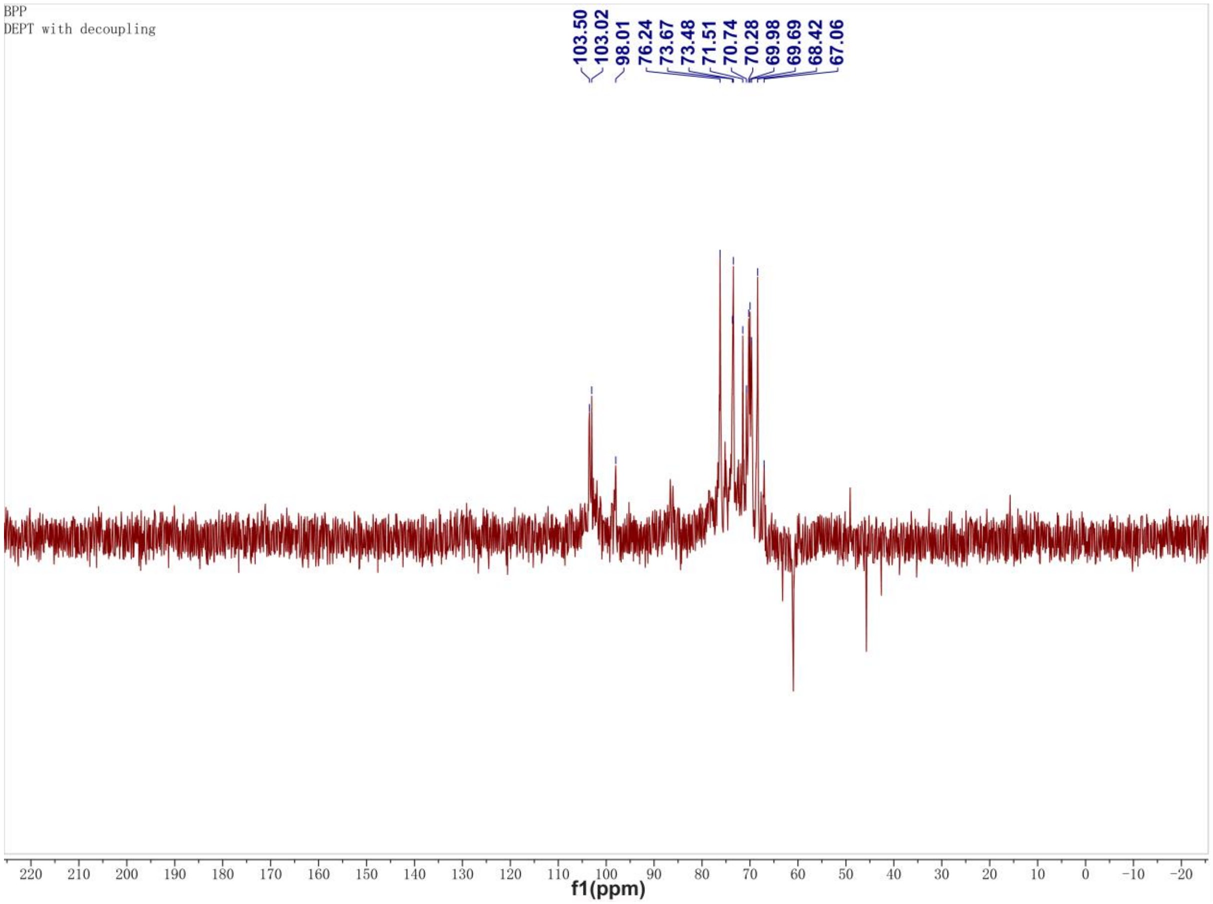

Figure S3: DEPT-135 spectra of BPP;

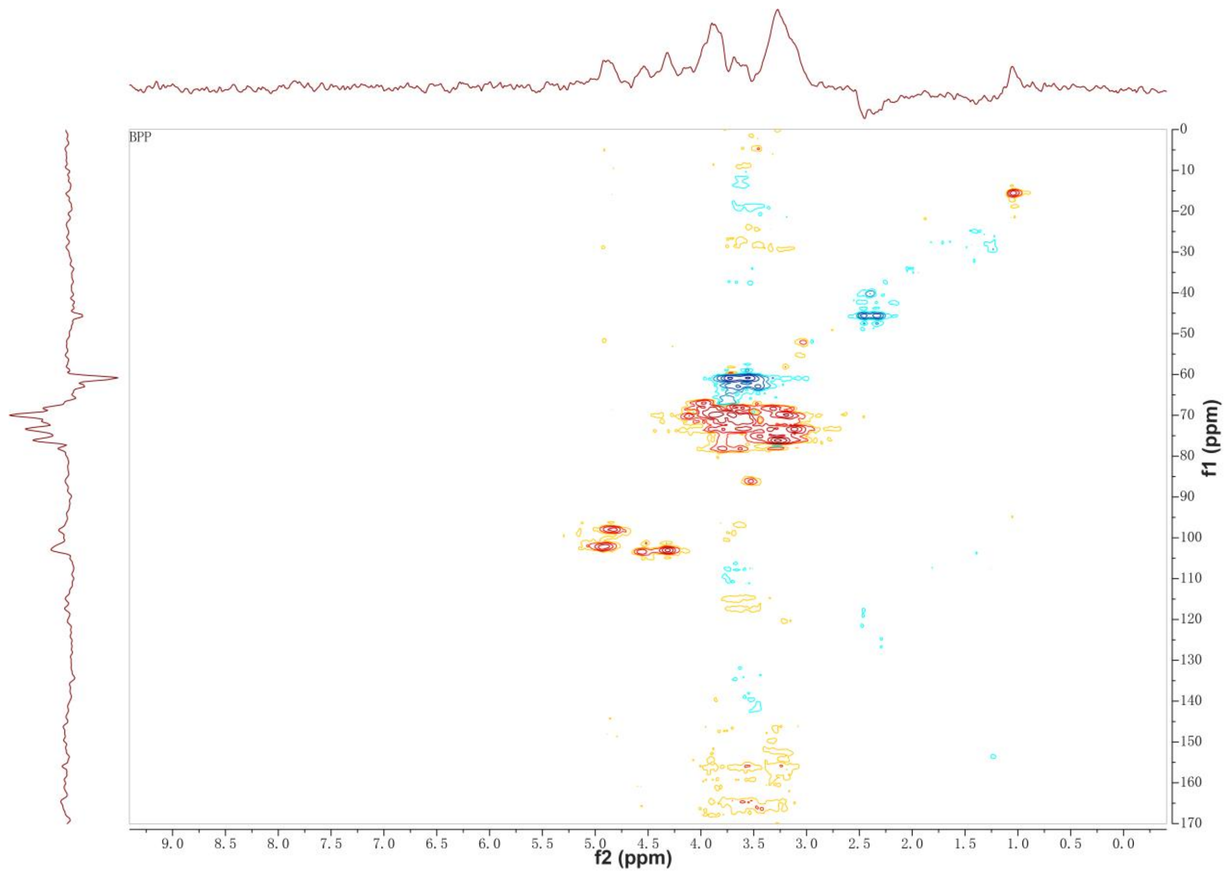

Figure S4: HSQC spectra of BPP;

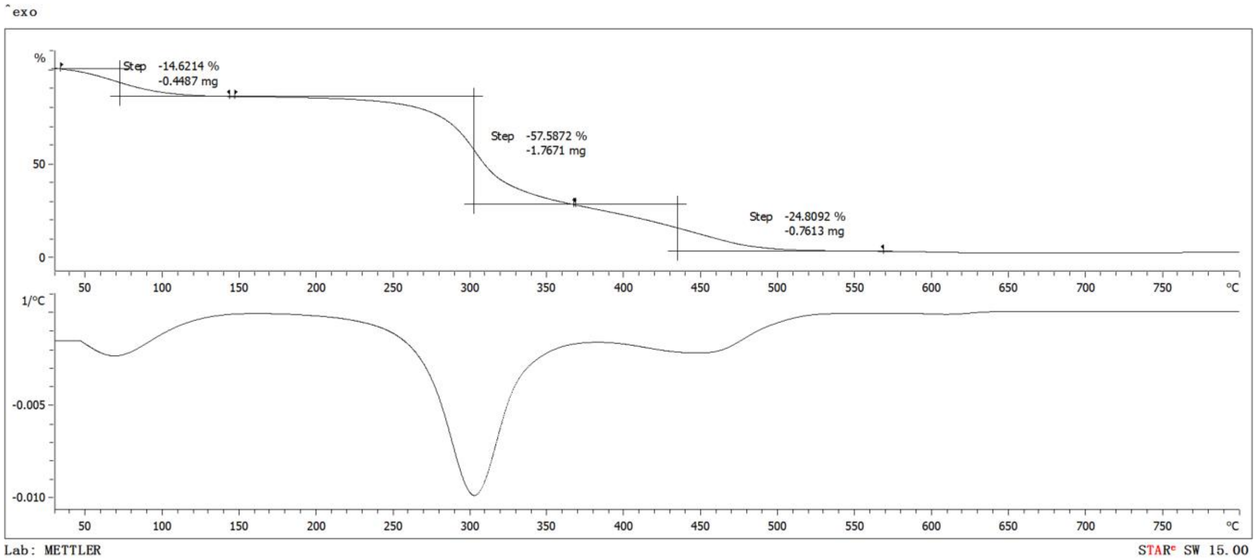

Figure S5: TG analysis of BPP under atmosphere at a heating rate of 10 K/min

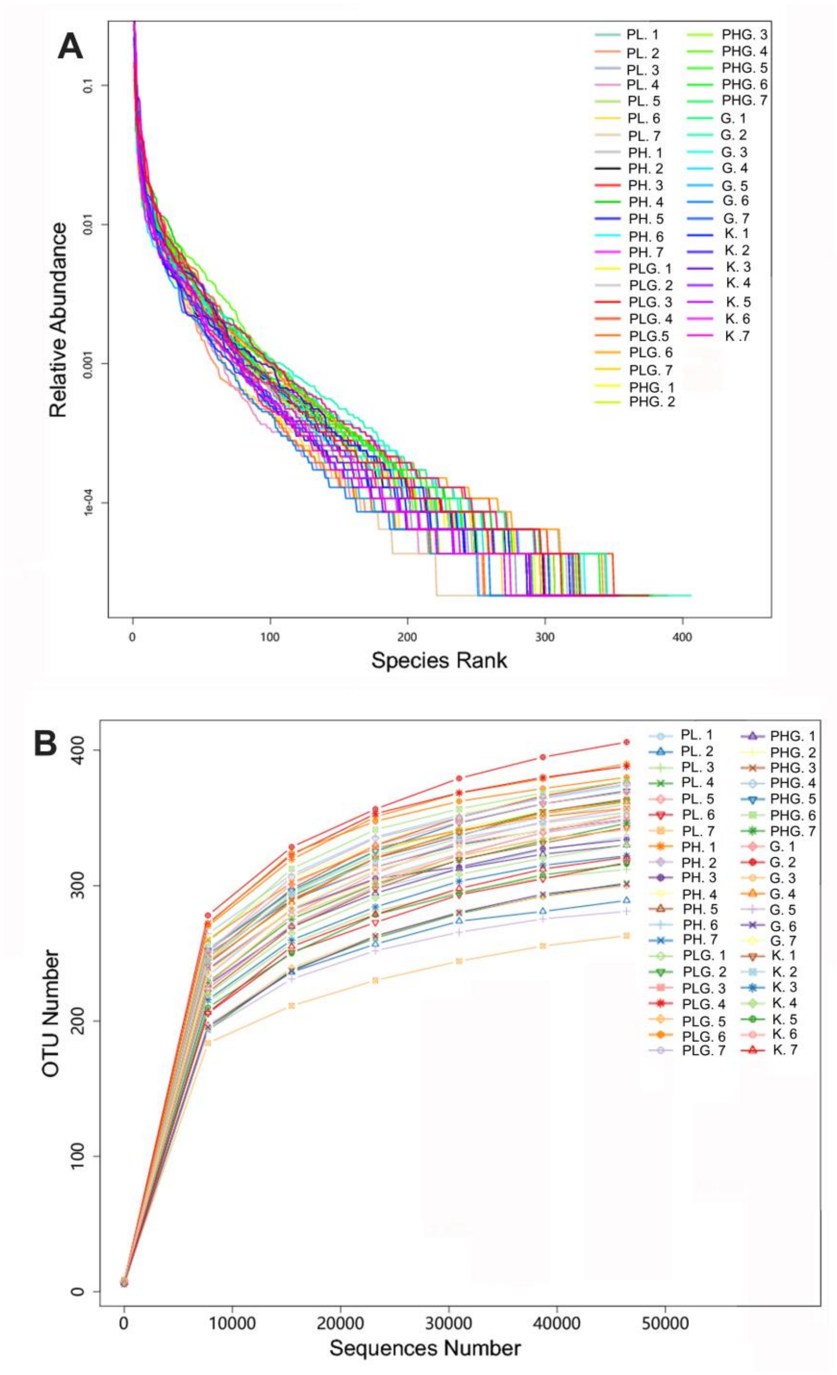

Figure S6: KEGG metabolic pathways at the second level

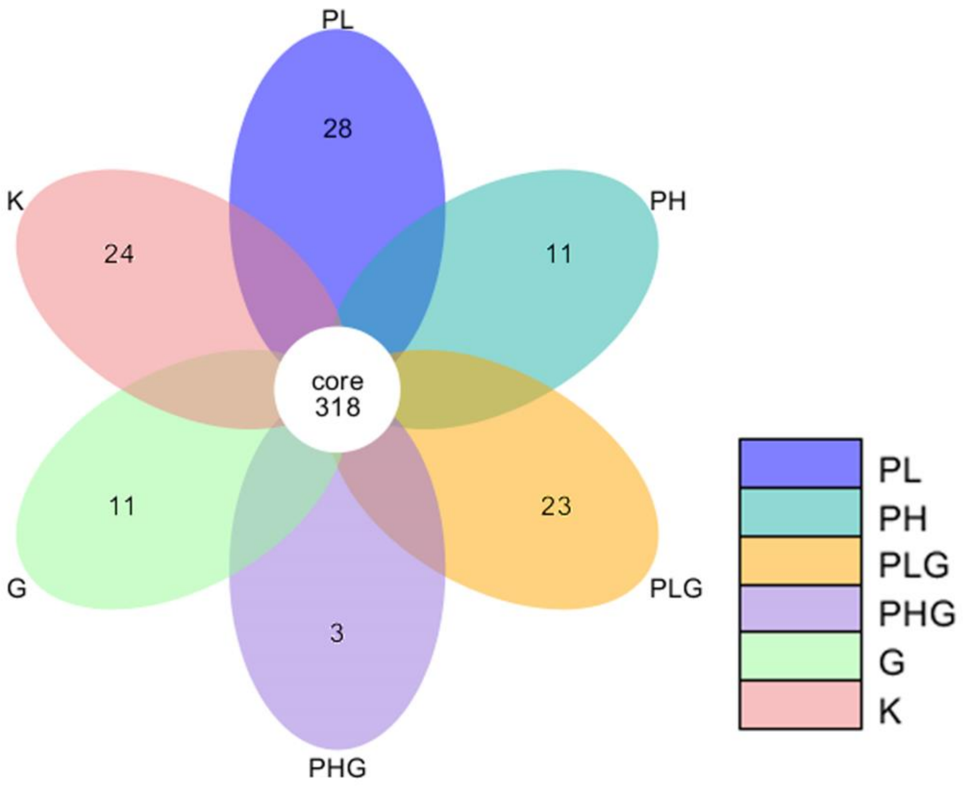

Figure S7: The flower figure OTUs distribution of flora with different treatments
